# Tryptase is involved in the development of early ventilator-induced pulmonary fibrosis in sepsis-induced lung injury

**DOI:** 10.1186/s13054-015-0878-9

**Published:** 2015-04-03

**Authors:** Jesús Villar, Nuria E Cabrera-Benítez, Francisco Valladares, Sonia García-Hernández, Ángela Ramos-Nuez, José Luís Martín-Barrasa, Mercedes Muros, Robert M Kacmarek, Arthur S Slutsky

**Affiliations:** CIBER de Enfermedades Respiratorias, Instituto de Salud Carlos III, Monforte de Lemos 3-5, 28029 Madrid, Spain; Multidisciplinary Organ Dysfunction Evaluation Research Network, Research Unit, Hospital Universitario Dr. Negrin, Barranco de la Ballena, s/n, Las Palmas de Gran Canaria, 35010 Spain; Keenan Research Center for Biomedical Science, St. Michael’s Hospital, 30 Bond Street, Toronto, ON M5B 1W8 Canada; Department of Anatomy, Pathology and Histology, University of La Laguna, Campus de CC. de la Salud, 38071 Tenerife, Spain; Department of Clinical Biochemistry, Hospital Universitario NS de Candelaria, Ctra. Gral. del Rosario, 145, Santa Cruz de Tenerife, 38010 Spain; Department of Respiratory Care, Massachusetts General Hospital, 55 Fruit Street, Boston, MA 02114 USA; Department of Anesthesia, Harvard University, 75 Francis Street, Boston, MA 02115 USA; Interdepartmental Division of Critical Care Medicine, University of Toronto, 585 University Avenue, Toronto, ON M5G 2N2 Canada

## Abstract

**Introduction:**

Most patients with sepsis and acute lung injury require mechanical ventilation to improve oxygenation and facilitate organ repair. Mast cells are important in response to infection and resolution of tissue injury. Since tryptase secreted from mast cells has been associated with tissue fibrosis, we hypothesized that tryptase would be involved in the early development of ventilator-induced pulmonary fibrosis in a clinically relevant model of sepsis-induced lung injury.

**Methods:**

Prospective, randomized, controlled animal study using Sprague-Dawley rats. Sepsis was induced by cecal ligation and perforation. Animals were randomized to spontaneous breathing or two ventilatory strategies for 4 h: protective ventilation with tidal volume (VT) = 6 ml/kg plus 10 cmH_2_O positive end-expiratory pressure (PEEP) or injurious ventilation with VT = 20 ml/kg plus 2 cmH_2_O PEEP. Healthy, non-ventilated animals served as non-septic controls. We studied the following end points: histology, serum cytokine levels, hydroxyproline content, tryptase and proteinase-activated receptor-2 (PAR-2) protein level in lung homogenates, and tryptase and PAR-2 immunohistochemical localization in the lungs.

**Results:**

All septic animals developed acute lung injury. Animals ventilated with high VT had a significant increase of pulmonary fibrosis, hydroxyproline content, tryptase and PAR-2 protein levels compared to septic controls (*P* <0.0001). However, protective ventilation attenuated sepsis-induced lung injury and decreased lung tryptase and PAR-2 protein levels. Immunohistochemical staining confirmed the presence of tryptase and PAR-2 in the lungs.

**Conclusions:**

Mechanical ventilation modified tryptase and PAR-2 in injured lungs. Increased levels of these proteins were associated with development of sepsis and ventilator-induced pulmonary fibrosis early in the course of sepsis-induced lung injury.

## Introduction

Acute lung injury during the acute respiratory distress syndrome (ARDS) complicates a variety of clinical conditions and is associated with significant morbidity and mortality [1]. Sepsis, the most common cause of ARDS, promotes or interferes with mechanisms involved in tissue repair [2]. Mechanical ventilation (MV) is an essential life support for sepsis/ARDS patients and manipulation of the ventilator strategy is the only proven treatment for improving survival [3]. However, MV can cause or aggravate lung injury, an entity referred to as ventilator-induced lung injury (VILI) [4]. Experimental and clinical studies [3-6] have provided insights into the physiology of VILI, demonstrating that some patterns of MV result in pulmonary and systemic changes that mimic ARDS and sepsis [2-4]. Many ARDS patients survive the underlying disease but die with pulmonary fibrosis [7,8]. Recent reports suggest that sepsis may trigger the development of persistent fibrosis [9] and that VILI may be a major contributor to lung fibrosis [10,11].

The role of MV as an inciting factor for lung fibrosis is poorly understood. Pulmonary fibrosis appears to be an important determinant of mortality, regardless of the cause of ARDS [11,12]. Areas of fibrosis are adjacent to inflammation in the early exudative phase of ARDS [13-15]. A feature of lungs in patients with fibrotic lung disease is the increased number of mast cells [16] and it has been suggested that mast cells may support the continuation of the fibroproliferative process in patients with ARDS [17] by release of mediators. The most abundant product of mast cells is tryptase, a serine protease with pleiotropic biological activities [18]. Tryptases consist of α-tryptase and β-tryptase [19]. β-tryptase is the main isoenzyme expressed in human lung. Tryptase upregulates the expression of cytokines [19] and vascular endothelial growth factor (VEGF) [20]. We have previously reported that MV modulates the innate immune response by interfering with Toll-like receptors [6] and increases VEGF by activating the Wnt/β-catenin signaling pathways [10]. Tryptase is a potent stimulant of the synthesis of type I collagen by fibroblasts [21]. The mechanism by which tryptase exerts its effects is by activating a member of the protease-activated receptor (PAR) family, PAR-2 [22]. Currently, no published reports have examined the changes of tryptase and PAR-2 in the context of septic ARDS and VILI. We tested the hypothesis that tryptase content is modulated by MV and could contribute to the early development of pulmonary fibrosis in an experimental, clinically relevant animal model of sepsis-induced acute lung injury.

## Material and methods

This protocol was approved by the Animal Care Committee at the Hospital Universitario Dr. Negrín (CEEBA-003/10), in accordance with the European Commission Directive 2010/63/EU for animal experimentation. This study followed the ARRIVE guidelines for reporting preclinical animal research [23].

### Animal preparation and experimental protocol

A total of 40 male Sprague-Dawley rats (300 to 350 g) were included. Animals were anesthetized with an intraperitoneal injection of ketamine hydrochloride (80 mg/kg) and xylazine (8 mg/kg) [24]. Animals were initially randomized to two groups: non-septic controls (n = 6) and septic (n = 34). Sepsis was induced by cecal ligation and perforation (CLP). CLP is considered the gold standard model for experimental sepsis [25]. A detailed description of this model is provided elsewhere [26]. All septic animals received 10 ml normal saline subcutaneously immediately after CLP for postoperative fluid resuscitation. Eighteen hours after CLP, the peritoneal cavity was reopened in surviving animals and the cecum was excised and removed distal to the ligature. We then washed the peritoneal cavity with 20 ml warm, normal saline, and gently squeezed the abdomen several times. After closing the abdomen, each animal received 10 ml normal saline subcutaneously for fluid resuscitation throughout the experimental period. Then, animals were randomized to spontaneous breathing or two strategies of MV for 4 h: (i) protective MV using a tidal volume (VT) of 6 ml/kg plus 10 cmH_2_O of positive end-expiratory pressure (PEEP) or (ii) injurious MV using a VT of 20 ml/kg plus 2 cmH_2_O PEEP. We used a VT of 20 ml/kg because it produces regional lung stretch comparable to that experienced by ARDS patients in nondependent areas of the lung [26,27]. In previous pilot experiments, we found that the minimal level of overdistension in healthy rats causing an identifiable lung injury is 20 ml/kg. Healthy, anesthetized, non-ventilated animals served as non-septic controls.

In animals assigned to MV, we performed a tracheotomy using a 14-G Teflon catheter. Animals were then paralyzed (1 mg/kg pancuronium bromide), connected to a time-cycled, volume-limited rodent ventilator (Ugo Basile, Varese, Italy) and placed on a temperature-controlled table to maintain body temperature at 37°C. Fraction of inspired oxygen (FiO_2_) was 0.60 in both MV groups. Ventilator rate was set at 90 cycles/min and 30 cycles/min in the low and high VT groups, respectively, to maintain constant minute ventilation and comparable partial pressure of carbon dioxide (PaCO_2_), based on our previous experiments with the same animal model and identical setup [24]. Animals were monitored noninvasively to minimize the possibility of triggering an inflammatory response, after establishing a protocol that provided hemodynamic stability and comparable arterial blood gases. Animals were ventilated supine on a restraining board inclined 20° from the horizontal, and received intermittent intraperitoneal boluses of anesthetic and paralytic agents during the 4-h experimental period. Peak airway pressures were continuously monitored. Oxygen saturation was continuously measured using a pulse oxymeter applied to the rat's tongue.

### Histological examination

At the end of the 4-h observation and ventilation period, animals were sacrificed by supplemental pentobarbital (10 mg/kg), followed by exsanguination by cutting the abdominal vessels. The lungs were removed from the chest, the trachea cannulated, and the right lung was fixed by intratracheal instillation of 3 ml 10% formalin. Two pathologists (FV, SGH) blinded to group identity examined random sections of the lung from each animal with particular reference to alveolar and interstitial damage defined as the presence of cellular inflammatory infiltrates, pulmonary edema, disorganization of lung parenchyma, alveolar rupture and hemorrhage. A semiquantitative morphometric analysis of lung injury was performed by scoring from 0 to 4 (none, mild, moderate, severe, very severe) for each parameter. A total histological lung injury score [13] was obtained by adding the individual scores in every animal and averaging the total values in each group.

Histological features of lung fibrosis were evaluated with Masson-Goldner staining under light microscopy (Nikon Optiphot, Tokyo, Japan) and photographed with a Nikon Digital DS-5 M camera. Three longitudinal and random slides from each animal were used for analysis according to the method by Ashcroft *et al*. [28]. Each successive field was individually assessed for fibrosis using a semiquantitative score as follows: 0 = normal lung; 1 = minimal fibrous thickening of alveolar or bronchiolar walls; 2 = moderate thickening of walls without obvious damage to lung architecture; 3 = increased fibrosis with definite damage to lung structure and formation of fibrous bands or small fibrous masses; 4 = severe distortion of structure and large fibrous areas; 5 = total fibrous obliteration of the field. After examining all sections, a total score was calculated for each animal.

### Cytokine serum levels

At the end of the 4-h experimental period, 2 ml of blood were collected from the first six surviving rats in each group by cardiac puncture. After centrifugation for 15 min at 3,000 rpm, sera were divided into aliquots and frozen at −80°C. Tumor necrosis factor alpha (TNF-α) and interleukin 6 (IL-6) protein concentrations were measured by commercially available immunoassays (Cytoscreen, Biosource International, Camarillo, CA, USA) and performed according to the manufacturer’s specifications using an ELx800 NB Universal Microplate Reader (BioTek Instruments, Winooski, VT, USA). TNF-α and IL-6 concentrations are expressed as pg/ml.

### Hydroxyproline assay

Collagen synthesis is determined by a hydroxyproline assay [20]. Lung samples were homogenized in 2 ml phosphate-buffered saline (PBS), and 1 ml aliquot was hydrolyzed with 6 N HCl at 110°C for 18 h following the Woessner method [29]. Then, 25 μL aliquots were added to 1 ml of 1.4% chloramine T (Sigma-Aldrich, St Louis, MO, USA), 10% *2*-propanol, and 0.5 M sodium acetate, pH 6.0. After 20 min of incubation at room temperature, 1 ml Erlich's solution (1 M *p*-dimethyl-amino-benzaldehyde in 70% 2*n*-propanol and in 20% perchloric acid) was added, followed by a15-min incubation at 65°C. Samples were read in a spectrophotometer (ELx800 Absorbance Microplate Reader, BioTek Instrument, Luzen, Switzerland). The amount of hydroxyproline was determined against a standard curve. Data are expressed as μg/g of lung tissue.

### Immunoblotting

Left lungs from all experimental groups were excised, washed with saline, frozen in liquid nitrogen, and stored at −80°C for subsequent protein extraction and immunoblotting. Lungs were sampled in multiple areas, homogenized, and proteins were extracted by centrifugation at 14,000 rpm for 5 min at 4°C. Protein concentration was determined using the Bio-Rad DC Protein Assay (Bio-Rad Laboratories, Hercules, CA, USA). The supernatant (30 μg protein) was mixed with equal volume of 2 × SDS sample buffer and boiled for 5 min. Proteins were separated by SDS-polyacrylamide gel electrophoresis and transferred onto PVDF membranes. Blots were blocked for 1 h with 10% (w/v) dry non-fat milk in Tris-buffered saline (TBS)-Tween and probed with rabbit polyclonal primary anti-tryptase and anti-PAR-2 antibodies (Santa Cruz Biotechnology, Santa Cruz, CA, USA), followed by a goat anti-rabbit immunoglobulin G (IgG)-horseradish peroxidase (HRP) as secondary antibody (Santa Cruz Biotechnology). Blots were then stripped and reprobed with a β-actin antibody (Cell Signaling Technology, Danvers, MA, USA) to confirm equal protein loading. Quantification of blots was performed using the Scion Image software package (Scion Corp., Frederick, MD, USA).

### Immunohistochemistry

Lungs were fixed in 4% formaldehyde, paraffin embedded, sliced into 3-μm sections, deparaffinized, and antigen unmasked in citrate buffer solution (0.01 M pH 6.0). Then, endogenous peroxidase activity was blocked for 10 min at room temperature with 0.3% H_2_O_2_. Slides were incubated with rabbit primary anti-tryptase and anti-PAR-2 antibodies (Santa Cruz Biotechnology) in PBS for 1 h and with biotinylated anti-rabbit IgG secondary antibodies for 10 min at room temperature, and detected by the peroxidase-conjugated avidin-biotin complex reaction with 3-amino-9-ethylcarbazole AEC+/substrate chromogen (Dako, Hamburg, Germany). Sections were then counterstained with Mayer’s hematoxylin (Dako). Images were obtained using an Olympus (BX50) microscope (Olympus, Hamburg, Germany) and an Olympus digital camera.

### Statistical methods

For sample size calculations, we estimated a minimum of six animals per each subgroup to detect an absolute increase of 50% in the levels of fibrotic markers in the high-VT group, with an alpha = 0.05 and power >0.80. Data are expressed as mean ± standard deviation (SD). Groups were compared using a one-way analysis of variance followed by Bonferroni *post hoc* testing where appropriate. A two-sided *P* value <0.05 was considered statistically significant.

## Results

### Outcome and pathological evaluations

A total of seven rats died after CLP. The remaining 27 septic animals were randomly allocated to three groups (n = 9 in each group). Within the 4-h observational period, three septic animals died in the non-ventilated, spontaneous breathing group. No animals ventilated with protective MV died; one animal ventilated with high VT died. Only data from animals surviving the 4-h experimental period were analyzed. At the end of the 4-h MV period, peak inspiratory pressure was 28 ± 2 cmH_2_O in the high-VT group and 20 ± 2 cmH_2_O in the low-VT group (*P* <0.0001).

**Figure 1:**
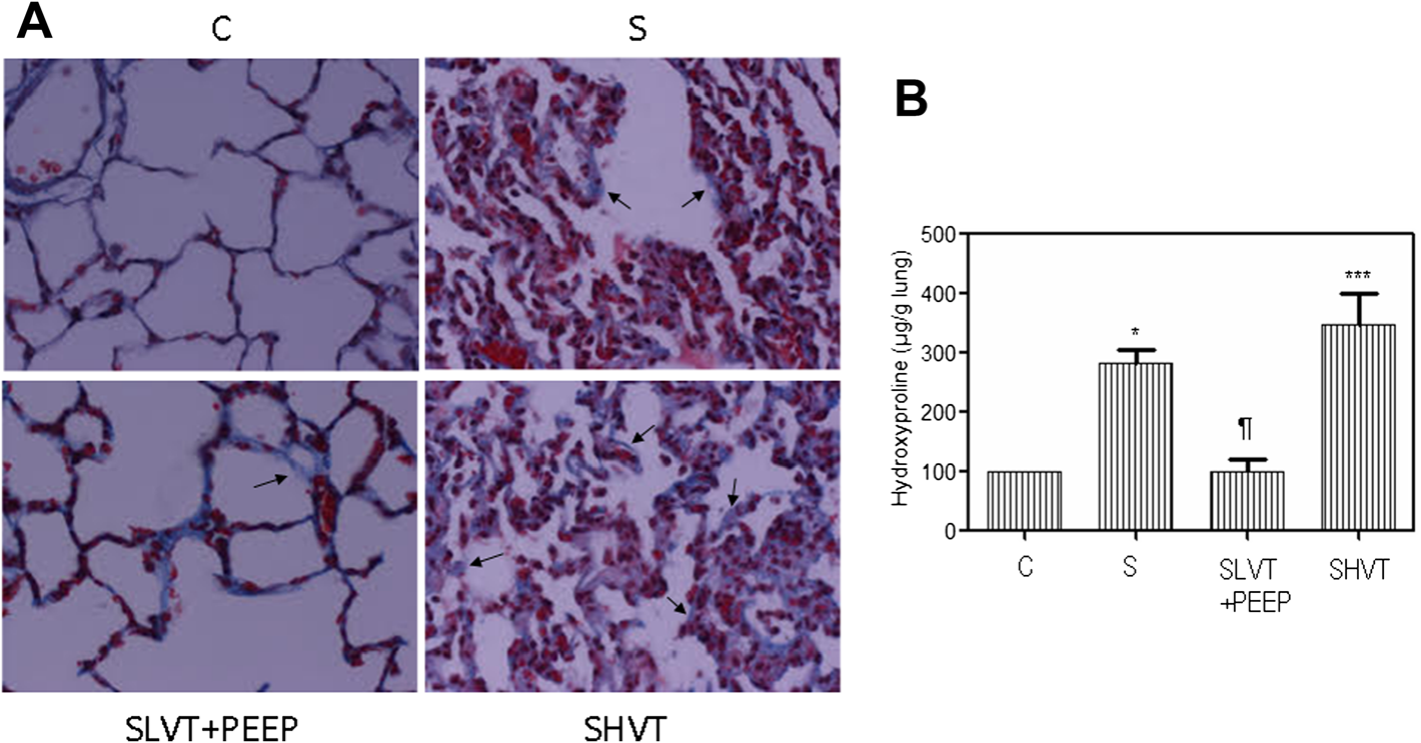
**Histological features of lung fibrosis and hydroxyproline content in lungs. (A)** Pathological features of lung fibrosis in rats with acute lung injury induced by sepsis and mechanical ventilation. Collagen deposition (see arrows) was marked in septic, non-ventilated and high-VT ventilated lungs (Masson-Goldner, ×400 magnification). **(B)** Hydroxyproline content in the lungs. C: healthy, anesthetized, non-ventilated, spontaneous breathing; S: septic, anesthetized, non-ventilated, spontaneous breathing; SLVT + PEEP: septic, ventilated with low tidal volume plus positive end-expiratory pressure (PEEP) of 10 cmH_2_O; SHVT: septic, ventilated with high tidal volume. Six animals were quantified in each group. ^¶^ *P* <0.01 vs. S; ^*^ *P* <0.01 vs. C; ^***^ *P* <0.0001 vs. SLVT + PEEP. VT, tidal volume.

Histological examination of septic lungs revealed the presence of acute lung injury and pulmonary fibrosis (Figure 1A). Septic animals ventilated with high VT had the highest histological injury scores (14.9 ± 1.5), whereas CLP animals ventilated with low VT had a lower histological injury score than non-ventilated CLP animals (5.5 ± 0.6 vs. 10.1 ± 1.0) (*P* <0.0001). The high-VT MV group had the highest lung fibrosis score (3.8 ± 0.3 vs. 2.3 ± 0.3 in septic, non-ventilated animals, *P* <0.001). By contrast, low-VT MV attenuated the degree of lung fibrosis (1.4 ± 0.2) (*P* <0.001).

### Hydroxyproline content

Sepsis increased hydroxyproline levels (284 ± 38 μg/g) in the lungs when compared to healthy controls (*P* <0.01). Hydroxyproline content was higher in animals ventilated with high VT (347 ± 94 μg/g) than with low VT (99 ± 35 μg/g) (*P* <0.0001) (Figure 1B).

### Serum cytokines

**Figure 2:**
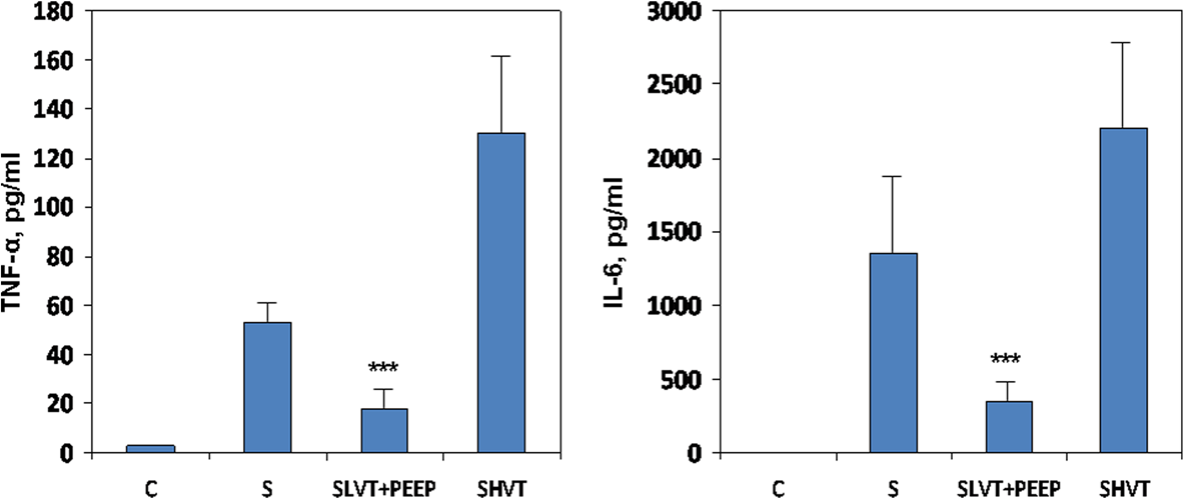
**Serum levels of tumor necrosis factor-alpha (TNF-α) and interleukin (IL)-6 in non-ventilated and mechanically ventilated septic animals.** C: healthy, anesthetized, non-ventilated, spontaneous breathing; S: septic, anesthetized, non-ventilated, spontaneous breathing; SLVT + PEEP: septic, ventilated with low tidal volume plus positive end-expiratory pressure (PEEP) of 10 cmH_2_O; SHVT: septic, ventilated with high tidal volume. Eight animals were quantified in each group. ^***^ *P* <0.0001 vs. S and SLVT + PEEP.

Systemic levels of cytokines were higher in high-VT MV septic animals (n = 8) than in low-VT animals (n = 9): TNF-α 130 ± 35 vs. 18 ± 9 pg/ml, *P* <0.0001; IL-6 2,209 ± 618 vs. 344 ± 98 pg/ml, *P* <0.0001, respectively. In non-ventilated septic animals (n = 6), TNF-α was 53 ± 10 pg/ml and IL-6 was 1,353 ± 542 pg/ml (Figure 2).

### Tryptase and PAR-2 protein levels and immunohistochemical staining

**Figure 3:**
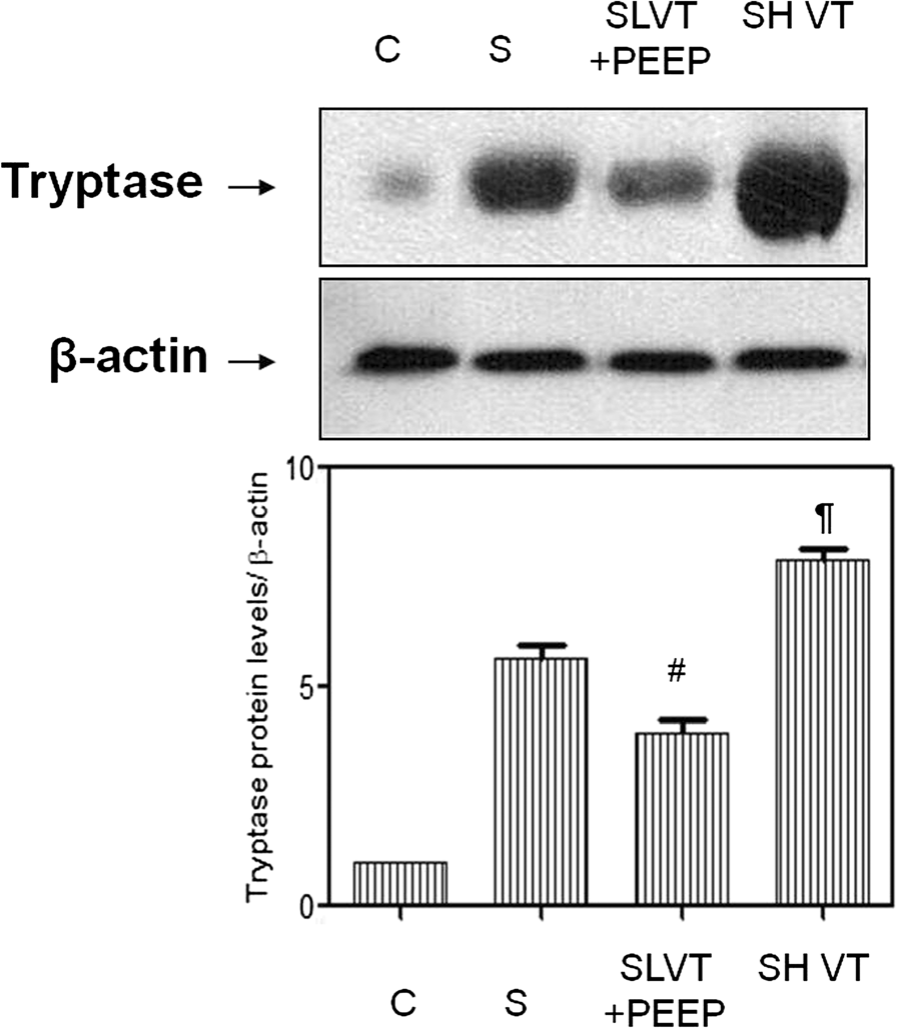
**Western blot analysis of tryptase.** The upper panel is a representative gel for each experimental group. The lower panel represents the relative optical density of tryptase normalized against β-actin. C: non-septic, anesthetized, non-ventilated, spontaneous breathing; S: septic, anesthetized, non-ventilated, spontaneous breathing; SLVT + PEEP: septic, ventilated with low tidal volume plus positive end-expiratory pressure (PEEP) of 10 cmH_2_O; SHVT: septic, ventilated with high tidal volume. Six animals were quantified in each group. ^*#*^ *P* < 0.01 vs. S; ^¶^ *P* <0.001 vs. S.

**Figure 4:**
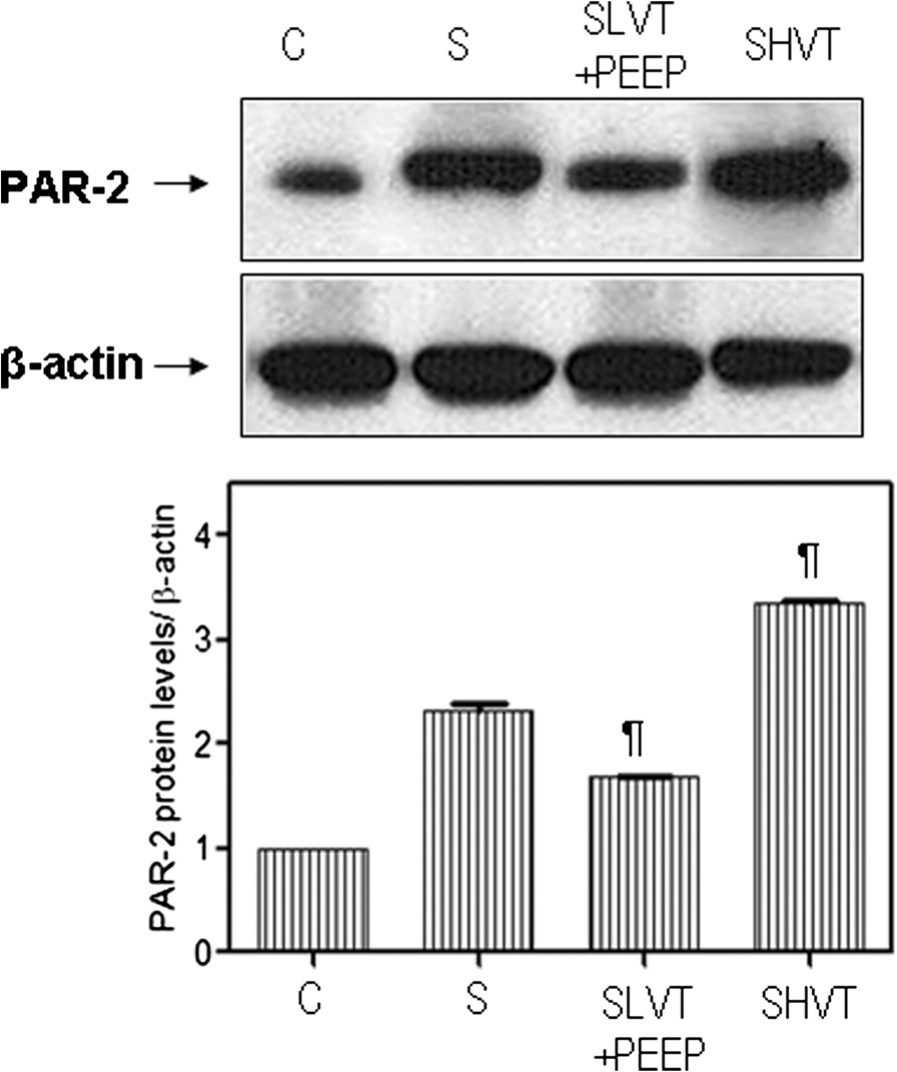
**Western blot analysis of PAR-2.** The upper panel is a representative gel for each experimental group. The lower panel represents the relative optical density of tryptase normalized against β-actin. C: non-septic, anesthetized, non-ventilated, spontaneous breathing; S: septic, anesthetized, non-ventilated, spontaneous breathing; SLVT + PEEP: septic, ventilated with low tidal volume plus positive end-expiratory pressure (PEEP) of 10 cmH_2_O; SHVT: septic, ventilated with high tidal volume. Six animals were quantified in each group. ^¶^ *P* <0.001 vs. S.

Tryptase and PAR-2 protein levels increased in all septic groups. The highest tryptase and PAR-2 protein levels were found in the lungs of septic animals ventilated with high VT (7.89 ± 0.44 vs. 5.64 ± 0.53 in septic, non-ventilated animals, *P* <0.001 for tryptase; 3.36 ± 0.04 vs. 2.32 ± 0.12, *P* <0.001 for PAR-2) (Figures 3 and 4).

**Figure 5:**
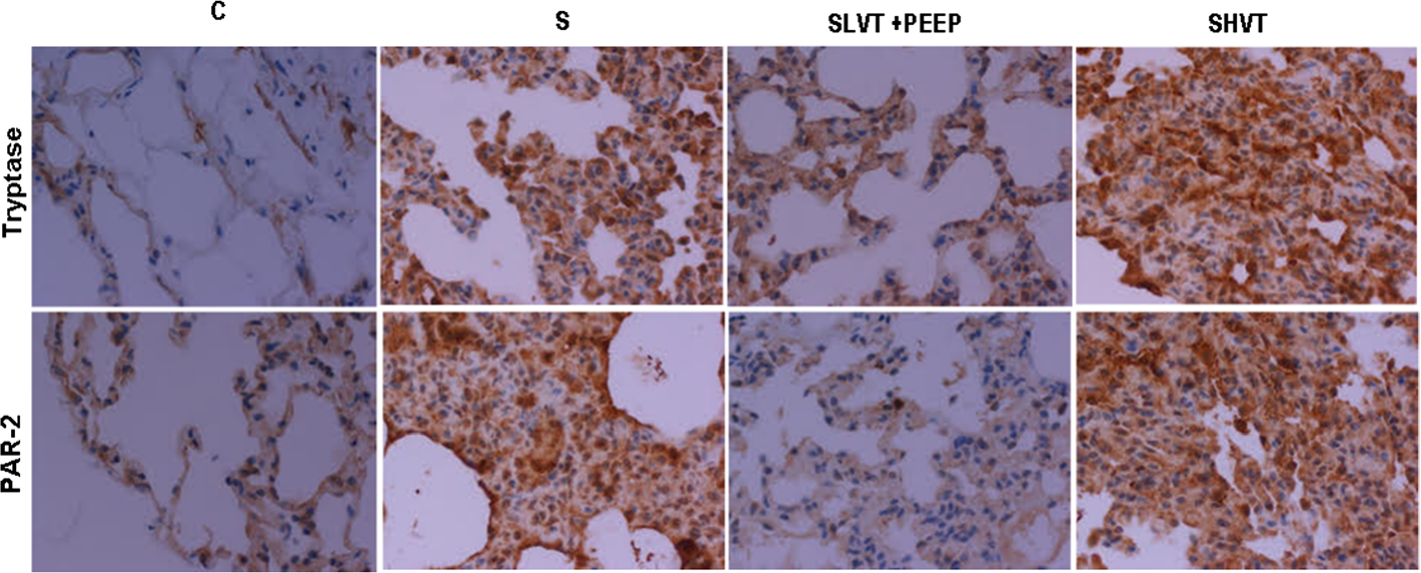
**Immunohistochemical localization of tryptase and PAR-2.** Brown color staining indicates positive staining and blue/violet indicates nuclei counterstained with hematoxylin. Tryptase and PAR-2 staining increased markedly in high-VT lungs. C: non-septic, anesthetized, non-ventilated, spontaneous breathing; S: septic, anesthetized, non-ventilated, spontaneous breathing; SLVT + PEEP: septic, ventilated with low tidal volume plus positive end-expiratory pressure (PEEP) of 10 cmH_2_O; SHVT: septic, ventilated with high tidal volume. ×400 magnification. PAR-2, protease-activated receptor-2; VT, tidal volume.

Tryptase and PAR-2 proteins (Figure 5) were predominantly expressed in mast cells and fibroblasts respectively. Control, healthy animals had low staining for tryptase and PAR-2. There was an intense expression of these peptides in the lungs of the high-VT group.

## Discussion

To our knowledge, this is the first report to provide evidence that the application of MV modifies tryptase and PAR-2 levels in the septic injured lung. Our main findings are: (i) protein content of tryptase of septic lungs after a short period of injurious MV was associated with a more than threefold increase of hydroxyproline compared to protective ventilation; and (ii) an injurious MV strategy increases pulmonary tryptase and collagen content in the context of sepsis-induced acute lung injury. Our findings support the hypothesis that tryptase and collagen lung content is modulated by MV and could contribute to the early development of pulmonary fibrosis in an experimental, clinically relevant animal model of sepsis-induced acute lung injury.

MV causes injury not only by structural disruption of the lung but also by inducing the release of mediators that can worsen lung damage and may cause multiorgan dysfunction [2]. Alveolar overdistension and cyclic recruitment and collapse of unstable lung units are the most prevalent biophysical mechanisms mediating VILI [30]. The importance of minimizing overdistension is supported by a compelling physiological rationale and by strong clinical evidence [3]. Given the reduced volume of ARDS lungs [31], low-VT ventilation plus moderate-to-high levels of PEEP contribute to lung protection as both mediate reductions in shear/strain stresses by distinct mechanisms [30]. MV has been shown to activate extracellular matrix elements such as collagen and proteoglycan [32]. Collagen is the most abundant stress-bearing component of the parenchymal tissue and plays a key role in determining the mechanical properties and cellular responses to injury [33]. The amount of collagen in the lung is tightly regulated to ensure a strict balance between synthesis and degradation. Human studies suggest that collagen content in the lung is a key determinant of active fibroproliferation or normal repair [34]. There is evidence of fibrotic changes in the earliest stages of ARDS [11,14,15]. Any alteration in the collagen content in the ARDS lung may contribute to cellular abnormalities and fatal outcome [8,34]. We have recently reported that the WNT/β-catenin signaling, a pathway known to induce fibroblast activation and collagen synthesis [35], is activated very early in sepsis-induced acute lung injury [36].

The exact mechanism regulating tryptase activity is unknown. Release of tryptase, proteoglycans, and cytokines from mast cells (known as degranulation), may be induced by physical and immune mechanisms [37]. Tryptase-containing mast cells from the lung are found mainly in alveolar walls. PAR-2, the specific cell surface receptor for tryptase [38], is expressed by airway epithelial and smooth muscle cells, endothelial and vascular smooth muscle cells, terminal bronchial epithelium, type II cells and mast cells within the respiratory tract [39]. Mast cells induce fibroblasts to proliferate and synthesize type I collagen. Mast cells are difficult to locate in routinely processed lung specimens because they often lose their characteristics staining properties with formalin fixation. However, they can be identified using immunohistochemical localization of tryptase [40].

The term ‘fibroproliferative’ has been conventionally applied only to ‘late-phase ARDS’, but pulmonary fibroproliferation can occur early in ARDS and is correlated with outcome [15,34]. Although in our animal model the exudative stage of acute lung injury and VILI is dominated by the presence of edema and inflammatory infiltrates [6], increased collagen was present in all lung samples at 18 h after CLP and increased further after 4 h of high-VT MV, confirming our previous results [10,13]. In lung tissues from 17 ARDS patients, Liebler *et al.* [17] found that 80% of samples from the early exudative stage exhibited increased number of myofibroblasts, and 50% had increased number of procollagen type I-producing cells. Ichikado *et al*. [15] evaluated 85 ARDS patients using high-resolution computed tomography within the first day of ARDS diagnosis, and found that 47% of them had radiological areas indicative of fibroproliferation. Patients with less fibroproliferative changes on scans had a significantly lower mortality and more ventilator-free days than those with more extensive areas of fibroproliferation on day 1, despite the fact that both groups were ventilated with a mean VT of 8 ml/kg predicted body weight and 8 cmH_2_O of PEEP.

Our findings have two major clinical implications. First, alveolar overdistension is a driver for the development of pulmonary fibrosis since protective MV was able to attenuate the fibroproliferative response. We recently demonstrated that TGF-β and wingless-type integration site family, member 5A (WNT5A) activation are also important mechanisms involved in lung fibrosis after MV in murine models of acute lung injury and VILI [10,13,14]. We found that the degree of lung fibrosis was dependent on the severity of VILI in a two-hit *in vivo* mouse model of acid aspiration-induced lung injury followed by a MV strategy causing lung overdistension [14]. Second, it is plausible that pharmacological strategies aimed directly at attenuating the fibroproliferative response may enhance the transition to a normal repair process and, hopefully, improve survival of ARDS. Selective inhibition of lung tryptase or PAR-2 may be potential pharmacological targets to inhibit the early fibroproliferation that occurs by mechanical overdistension in ARDS. Akers *et al.* [22] demonstrated that tryptase-induced fibroblast proliferation can be inhibited by proteolytic inhibitors, and that human or rat PAR-2 mimicked the proliferative effects of tryptase. Taken together, these data suggest that tryptase induces proliferation of lung fibroblast by activating PAR-2. Recently developed tryptase and PAR-2 antagonist compounds have been shown to inhibit the tryptase- and PAR-2-induced tissue responses [41,42], representing a novel therapeutic approach for preventing and treating ventilator- and ARDS-induced pulmonary fibrosis. Further research in these areas is needed since the potential for tryptase and PAR-2 inhibitors to attenuate MV-induced fibrosis is unknown.

Our study has some limitations. First, although we showed that MV modulated tryptase/PAR-2 protein levels in the context of a clinically relevant model of sepsis-induced lung injury, our data do not confirm that this is a major pathway responsible for the MV-induced pulmonary fibrosis since we did not examine the effect of disrupting tryptase/PAR-2 protein levels by specific inhibitors or use tryptase/PAR-2-deficient animals. However, using a rat model of ARDS induced by intestinal ischemia-reperfusion injury, Gan *et al*. [43] found that the development of lung injury was accompanied with concomitant increases of tryptase and PAR-2 expressions in the lungs. They also demonstrated that by inhibiting tryptase, lung injury was reduced while activating mast cells further aggravated lung damage. In addition, other investigators have found that tryptase-deficient animals are less susceptible than their wild-type counterparts to lung injury and fibrosis under experimental conditions [44]. Also, in PAR-2 knockout mice, PAR-2 deficiency reduced the progression of liver fibrosis, hepatic collagen gene expression and hydroxyproline content [45]. Second, further mechanistic studies need to be done to elucidate the molecules and pathways involved in lung protection with regard to ARDS/fibrosis during low-VT ventilation. Finally, we have not examined whether PAR-2 could be activated by other proteases, such as coagulation factor Xa [46].

## Conclusions

Our experimental findings provide evidence for an association between acute lung injury, tryptase, PAR-2, and pulmonary fibrosis in VILI and sepsis-induced lung injury. Further studies are needed to fully address whether the attenuation or inhibition of tryptase and/or PAR-2 may offer a potential clinical therapeutic option in the setting of VILI and sepsis-induced ARDS.

## Key messages

- Changes in tryptase and collagen lung content during sepsis-induced acute lung injury are dependent on the mechanical ventilation strategy.
- Increased expression of tryptase and collagen induced by alveolar overdistension may be important events contributing to the development of ventilator-induced pulmonary fibrosis.
- Attenuation or inhibition of tryptase and/or PAR-2 may offer a potential clinical therapeutic option in the setting of VILI and sepsis-induced ARDS.

## Abbreviations

ARDS: acute respiratory distress syndrome
CLP: cecal ligation and puncture
FiO_2_: fraction of inspired oxygen
HRP: horseradish peroxidase
IgG: immunoglobulin G
IL-6: interleukin 6
MV: mechanical ventilation
PaCO_2_: partial pressure of carbon dioxide
PAR-2: protease-activated receptor-2
PBS: phosphate-buffered saline
PEEP: positive end-expiratory pressure
SD: standard deviation
TBS: Tris-buffered saline
TNF-α: tumor necrosis factor alpha
VEGF: vascular endothelial growth factor
VILI: ventilator-induced lung injury
VT: tidal volume
WNT5A: wingless-type integration site family, member 5A
